# 3D high content imaging: high level phenotypic quantification new opportunity for drug discovery

**Published:** 2011-01-10

**Authors:** Thierry Dorval, Regis Grailhe

**Affiliations:** 1 Cellular Differentiation, Institut Pasteur Korea, 696 Sampyeong-dong, Bundang-gu, Seongnam-si, Gyeonggi-do, 463-400, Korea; 2 Neurodegeneration and Applied Microscopy, Institut Pasteur Korea, 696 Sampyeong-dong, Bundang-gu, Seongnam-si, Gyeonggi-do, 463-400, Korea

## 

Advances in automated imaging microscopy allow fast acquisitions of multidimensional biological samples. Those microscopes open new possibilities for analyzing subcellular structures and spatial cellular arrangements. We present a 3D image analysis framework perfectly well suited for medium-throughput screening. Upon adaptive and regularized segmentation, followed by precise 3D reconstruction, we achieve automatic quantification of numerous relevant 3D descriptors related to the shape, texture, and fluorescence intensity of multiple stained subcellular structures. A global analysis of the 3D reconstructed scene shows additional possibilities to quantify the relative position of organelles. Implementing this methodology, we analyzed the subcellular reorganization of the nucleus, the Golgi apparatus and the centrioles occurring during the cell cycle. In addition, we quantified the effect of a genetic mutation associated with the early onset primary dystonia on the redistribution of torsinA from the bulk endoplasmic reticulum to the perinuclear space of the nuclear envelope. We show that the method enables the classification of various translocation levels of torsinA and opens the possibility for compound-based screening campaigns. Finally we present real applications of 3D cellular phenotype quantification in the screening context.

